# Formation of Complexes at Plasmodesmata for Potyvirus Intercellular Movement Is Mediated by the Viral Protein P3N-PIPO

**DOI:** 10.1371/journal.ppat.1000962

**Published:** 2010-06-24

**Authors:** Taiyun Wei, Changwei Zhang, Jian Hong, Ruyi Xiong, Kristin D. Kasschau, Xueping Zhou, James C. Carrington, Aiming Wang

**Affiliations:** 1 Southern Crop Protection and Food Research Centre, Agriculture and Agri-Food Canada, London, Ontario, Canada; 2 Department of Biology, The University of Western Ontario, London, Ontario, Canada; 3 College of Horticulture, Nanjing Agricultural University, Nanjing, People's Republic of China; 4 Center of Analysis and Measurement, Zhejiang University, Hangzhou, People's Republic of China; 5 Center for Genome Research and Biocomputing, Oregon State University, Corvallis, Oregon, United States of America; 6 Institute of Biotechnology, Zhejiang University, Hangzhou, People's Republic of China; University of California San Diego, United States of America

## Abstract

Intercellular transport of viruses through cytoplasmic connections, termed plasmodesmata (PD), is essential for systemic infection in plants by viruses. Previous genetic and ultrastructural data revealed that the potyvirus cyclindrical inclusion (CI) protein is directly involved in cell-to-cell movement, likely through the formation of conical structures anchored to and extended through PD. In this study, we demonstrate that plasmodesmatal localization of CI in *N. benthamiana* leaf cells is modulated by the recently discovered potyviral protein, P3N-PIPO, in a CI:P3N-PIPO ratio-dependent manner. We show that P3N-PIPO is a PD-located protein that physically interacts with CI *in planta*. The early secretory pathway, rather than the actomyosin motility system, is required for the delivery of P3N-PIPO and CI to PD. Moreover, CI mutations that disrupt virus cell-to-cell movement compromise PD-localization capacity. These data suggest that the CI and P3N-PIPO complex coordinates the formation of PD-associated structures that facilitate the intercellular movement of potyviruses in infected plants.

## Author Summary

Plant viral pathogens cause an estimated US$60 billion loss in crop yields worldwide each year. Potyviruses, accounting for ∼30% of known plant viruses, include many agriculturally important viruses. Despite their importance, the cell-to-cell spread of potyviruses remains poorly understood. Previous studies have shown that at early time points of infection, the virus-encoded CI protein, one of 11 known potyviral proteins, is associated with cone-shaped structures at plasmodesmata (PD) and is involved in viral cell-to-cell movement. In this paper, we show that a newly identified potyviral protein, P3N-PIPO, is a PD-located protein and directs the CI protein to PD, facilitating the deposition of the cone-shaped structures of CI at PD by interacting with CI protein. We demonstrate that the mutant of CI, which impairs potyviral cell-to-cell movement, loses its ability to accumulate at PD. We further reveal that P3N-PIPO utilizes the secretory pathway rather than the actomyosin motility system for trafficking to PD. Taken together, the data presented in this study suggest that CI and P3N-PIPO coordinates the formation of conical structure at PD for potyviral cell-to-cell spread.

## Introduction

The ability of plant viruses to cross the cell wall barrier between an infected and adjacent healthy cell is a prerequisite to establish systemic infection [1]–[6]. Cell-to-cell movement of viruses occurs through plasmodesmata (PD), a specialized intercellular organelle, unique to the plant kingdom [1], [2]. PD are structurally complex microchannels that cross the cell wall and establish cytoplasmic and endomembrane continuity between neighboring cells [1], [2], [7], [8]. PD allow small molecules to diffuse between cells and regulate the intercellular trafficking of macromolecules or macromolecular complexes such as virions and ribonucleoprotein complexes [1], [2], [7], [8]. Viral cell-to-cell movement through PD is mediated by virus-encoded factors termed movement protein (MP) [9], [10]. Based on the characteristics of their intercellular transport, plant viruses can be classified into several groups. One group, which includes tobamoviruses, encodes a single dedicated MP that associates with, and increases the size exclusion limits of, PD to allow transport of virions or viral genomes (nucleic acids) through the modified channel [11], [12]. The second group includes many plant viruses with icosahedral particles, and requires both the MP and coat protein (CP) for cell-to-cell movement. This group, which includes nepo- and comoviruses, encodes an MP that forms PD-associated tubules that traverse the cell wall and through which virions move [13], [14]. The third group includes several viruses with filamentous particles, such as potexviruses, that contain a set of three movement genes called the triple gene block. These genes encode proteins that are assumed to function coordinately, but without forming the tubular structure, to transport viral particles or genomes through PD [3], [4], [15].

**Figure 1:**
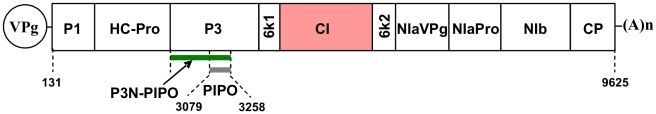
Schematic representation of the TuMV genome. The circle represents the genome-linked viral protein, VPg. Two short horizontal lines represent 5′ and 3′ untranslational region, respectively. The large box represents the long open reading frame (from nucleotides 131 to 9625). The mature proteins resulting from processing the large polyprotein are indicated as smaller boxes. PIPO (from nucleotides 3079 to 3258) derived from a frameshif on the P3 cistron is indicated as a shot grey bar. P3N-PIPO is indicated in green and CI in red. The poly(A) tail is shown as (A)n. For clarity, the relative sizes of the mature proteins are not drawn to scale.

The family *Potyviridae* consists of approximately 30% of known plant viruses, including many agriculturally important viruses, e.g., *Plum pox virus* (PPV), *Soybean mosaic virus* (SMV), *Turnip mosaic virus* (TuMV), *Tobacco etch virus* (TEV) and *Tobacco vein mottling virus* (TVMV) [16], [17]. Potyviruses contain a flexuous filamentous virion morphology composed of approximately 2000 copies of CP, and possess dimensions of 680–900 nm by 11–15 nm [17]. The potyviral genome is a single-stranded, positive-sense RNA of approximately 10 kilobases (kb), containing a long open reading frame (ORF) encoding a polyprotein that is cleaved into ∼10 mature proteins [18], [19]. They are, from the N terminus of the polyprotein, P1, helper component proteinase (HC-Pro), P3, 6K1, cyclindrical inclusion (CI), 6K2, viral genome-linked protein of nuclear inclusion protein a (NIa-VPg), proteinase domain of NIa (NIa-Pro), nuclear inclusion protein b (NIb), and CP (Figure 1). In addition, a small ORF encoding a recently identified ∼25 kDa protein, P3N-PIPO, is embedded in the P3 coding region as a plus 2 frameshift sequence (Figure 1) [20]. Of these 11 potyviral proteins, CI, CP, HC-Pro and VPg have been suggested to be involved in viral cell-to-cell movement [9], [21], [22]. Accumulating evidence indicates that HC-Pro and VPg are essential in other aspects of the infection process such as viral genome replication or suppression on host defense (RNA silencing) [23], [24], where as CP and CI are more likely to be MPs [9], [25]–[29]. Mutations in the conserved core region of CP abolish virion assembly and cell-to-cell movement, suggesting potyviruses likely move as virions [25], [26]. High-resolution ultrastructural analyses indicate that CI forms the cone-shaped structures at the cell periphery adjacent to PD [27]–[29]. CP and viral RNA are present in these PD-associated structures in infected cells [27], [28]. Moreover, substitutions affecting the N terminus of CI can result in loss of cell-to-cell movement without compromising virus replication at the single cell level [9]. These findings support the idea that potyvirus intercellular transport involves interactions between virus particles, CI structures and PD.

In this work, we present evidence that localization of CI to PD was modulated by potyviral protein P3N-PIPO in a ratio dependent manner. Trafficking of P3N-PIPO and CI to PD was via the secretory pathway. In addition, CI mutations that impaired virus cell-to-cell movement lost the ability to associate with PD. In combination with previous data, these results suggest that CI and P3N-PIPO coordinate the formation of the PD-associated conical structures for intercellular transport.

## Results

### Formation of TuMV CI punctate spots along the cell wall is mediated by P3N-PIPO in a CI:P3N-PIPO ratio-dependent manner

**Figure 2:**
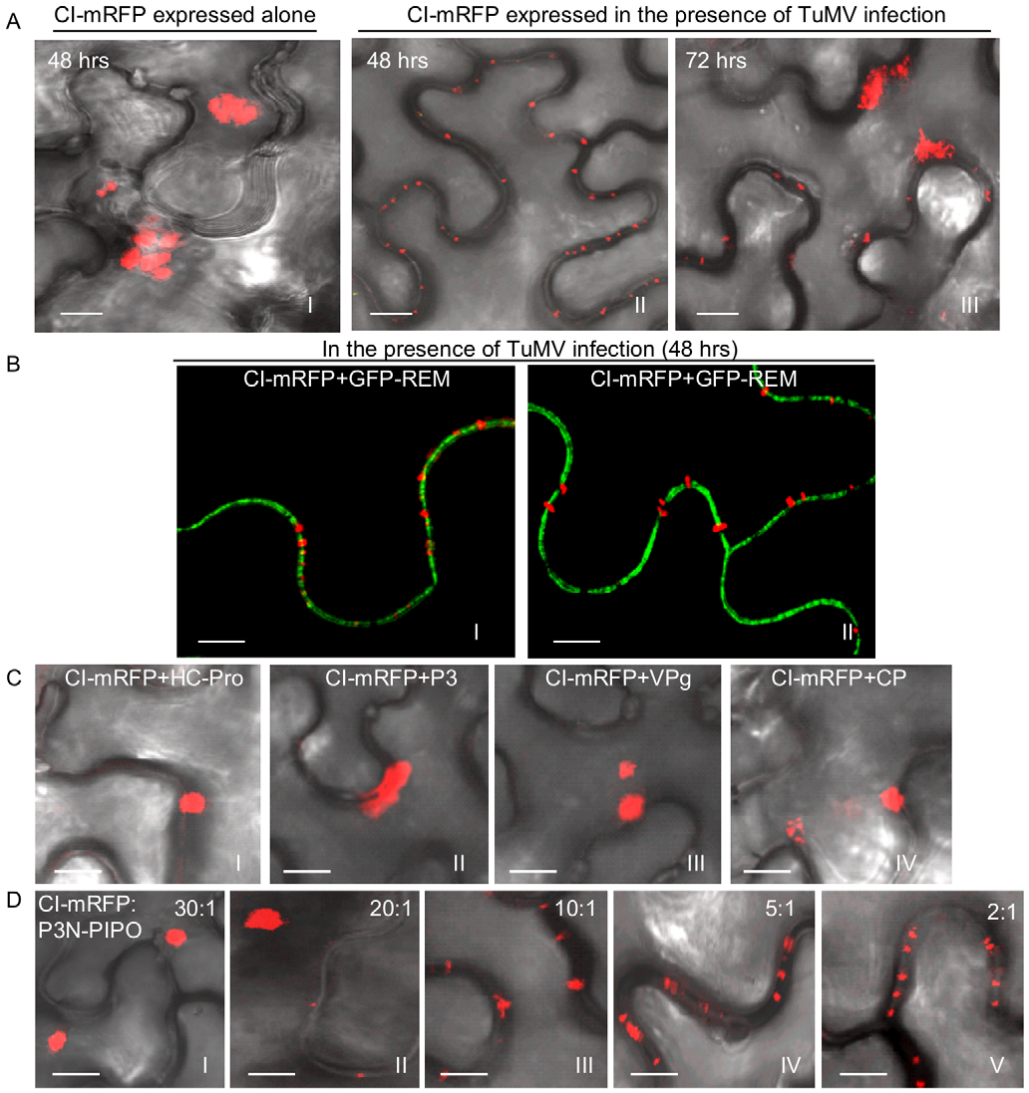
Subcellular localization of TuMV CI in *N. benthamiana* leaf cells. (A) Localization of CI-mRFP expressed alone in the leaf cells 48 hrs post-agroinfiltration (panel I) or in TuMV-infected leaf tissues 48 hrs (panel II) or 72 hrs (panel III) post-agroinfiltration. (B) Localization of CI-mRFP coexpressing with the plasma membrane marker GFP-REM (panels I, II) 48 hrs post-agroinfiltration. (C) Localization of CI-mRFP in the leaf cells coexpressing other viral proteins, i.e., HC-Pro (panel I), P3 (panel II), VPg (panel III) or CP (panel IV). Images were taken 48 hrs post-agroinfiltration. (D) Localization of CI-mRFP in the cells coexpressing P3N-PIPO. The ratio of agrobacterial culture mixtures containing plasmid CI to plasmid P3N-PIPO is indicated. Bars, 8 µm.

Previous high-resolution ultrastructural studies with several potyviruses have revealed that CI forms conical structures in close proximity to PD [27]–[29]. To study CI localization, *Agrobacterium*-mediated transient expression was used in this study. In *Nicotiana benthamiana* leaf cells, TuMV CI tagged with the momomeric red fluorescent protein (mRFP) formed thread-like structures that aggregated in the cytoplasm 48 hrs post-infiltration (Figure 2A, panel I). In contrast, CI-mRFP formed punctate spots along the cell wall in most cells (approximately 80%) when coexpressed with a recombinant infectious clone of TuMV tagged with the green fluorescent protein (GFP) (Figure 2A, panel II). These punctate bodies of CI-mRFP spanned walls of the two adjoining cells, appearing in patterns as PD [30]. At higher magnifications, the CI-mRFP fluorescence labeled thread-like structures of variable lengths (Figure 2B, panels I, II). The CI-mRFP structures resembled single or paired elongated bars that extended from the plasma membrane, which was labeled by REM-GFP (Figure 2B, panels I, II) [31]. Interestingly, by 72 hrs post-infiltration, many thread-like structures of CI-mRFP retracted from the cell wall and aggregated in the cytoplasm in the majority of infected cells (approximately 95%) (Figure 2A, panel III). Thus, association of the CI-mRFP with the cell wall appeared to be dependent on one or more virus-encoded factors in a transient fashion.

To determine if any TuMV protein facilitated targeting of the CI-mRFP to the cell wall, each viral protein (P1, HC-Pro, P3, P3N-PIPO, 6K1, 6K2, NIa, NIb and CP) was coexpressed individually with CI-mRFP. As illustrated using HC-Pro, P3, VPg and CP, coexpression with most TuMV proteins had no effect on the cytoplasmic aggregation of CI-mRFP (Figure 2C). In contrast, coexpression with P3N-PIPO resulted in the cell wall-associated punctate CI-mRFP (Figure 2D). Cell wall association was dependent on the CI-mRFP:P3N-PIPO ratio, with at least some CI-mRFP forming cytoplasmic aggregates at ratios of 10∶1 and higher (Figure 2D, Figure S1). These data suggest that CI is directed to cell walls through a dose-dependent interaction with, or is influenced by, P3N-PIPO.

### TuMV P3N-PIPO is a PD-located protein

**Figure 3:**
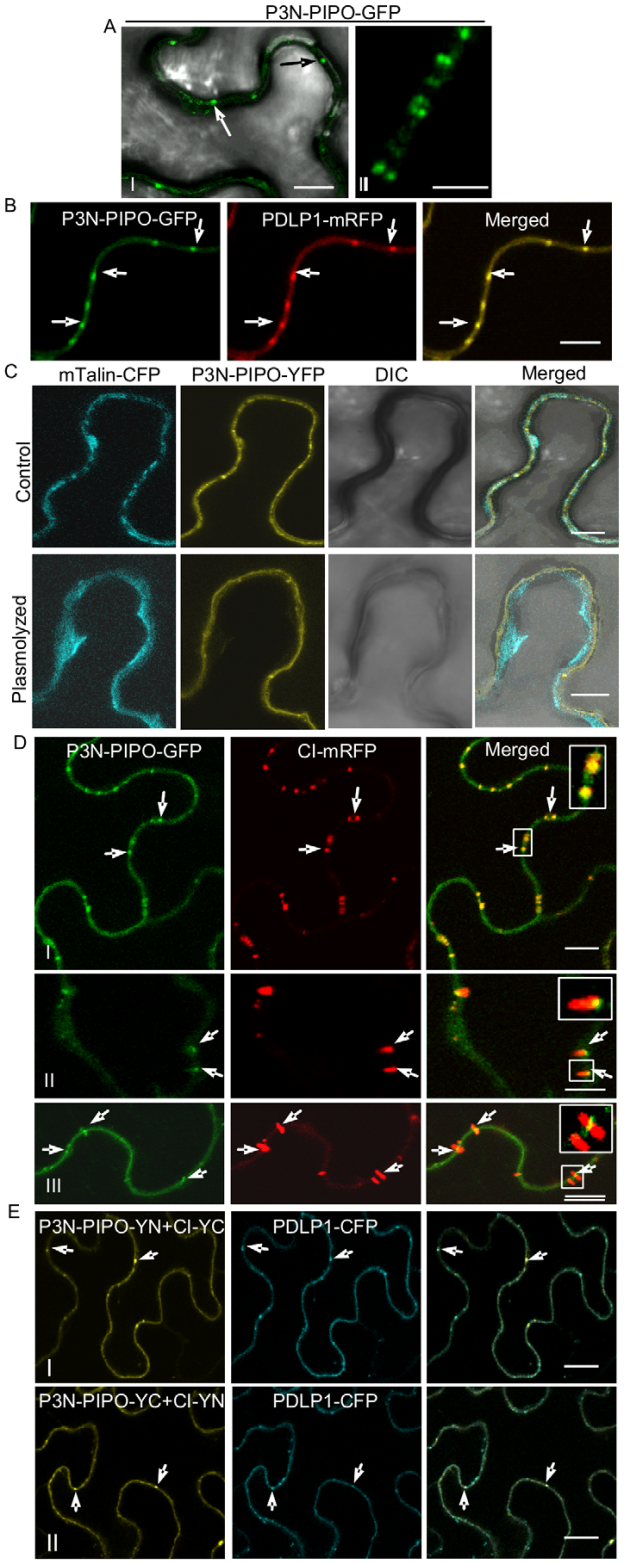
TuMV P3N-PIPO is a PD-localized protein and mediates the targeting of CI to PD in *N. benthamiana*. (A, panels I, II) Localization of P3N-PIPO-GFP transiently expressed in the cell treated 48 hrs post-agroinfiltration. Paired P3N-PIPO structures under a higher magnification (panel II). (B) Colocalization of P3N-PIPO-GFP with the PD marker PDLP1-mRFP. Arrows point to PD costained by P3N-PIPO-GFP and PDLP1-mRFP. (C) Cells coexpressing P3N-PIPO-YFP and mTalin-CFP as a cell membrane marker (control). Fluorescence of P3N-PIPO-YFP in plasmolyzed leaf tissue containing mTalin-CFP (plasmolyzed). DIC, Differential interference contrast. (D) Colocalization of P3N-PIPO with CI-mRFP 48 hrs (panel I) and 72 hrs (panels II, III) post-agroinfiltration. Arrows point to the PD-localized P3N-PIPO-GFP and CI-mRFP. Insets are the enlarged images of the areas in white boxes in the corresponding panels. (E) Interactions of TuMV CI and P3N-PIPO proteins *in vivo*. BiFC analysis (48 hrs post-agroinfiltration) was used to assess interactions in cells coexpressing CI-YC and P3N-PIPO-YN (panel I), CI-YN and P3N-PIPO-YC (panel II). Arrows indicate the strong BiFC fluorescence at PD costained by the PD marker, PDLP1-CFP. Bars, 8 µm.

To examine the intracellular distribution of P3N-PIPO *in planta*, P3N-PIPO-GFP was transiently expressed in *N. benthamiana* leaf cells via agroinfiltration. P3N-PIPO-GFP localized at the cell wall as punctate spots 48 hrs post-infiltration (Figure 3A, panel I). Under higher magnifications, paired punctate structures that spanned the adjoining cell walls were clearly evident (Figure 3A, panel II). P3N-PIPO-GFP was coexpressed with PDLP1-mRFP, a type I membrane plasmodesmatal protein [32]. P3N-PIPO-GFP and PDLP1-mRFP colabeled the punctate structures on the wall (Figure 3B), suggesting that P3N-PIPO is a PD-localized protein. To further confirm the localization of P3N-PIPO, P3N-PIPO tagged with the yellow fluorescent protein (YFP) and the actin marker mTalin tagged with the cyan fluorescent protein (CFP) were coexpressed in *N. benthamiana* leaf cells. In the unplasmolyzed cells, P3N-PIPO-YFP formed punctate spots on the cell wall, while mTalin-CFP fluorescence was evenly distributed throughout the cytoplasm compartment (Figure 3C, control). After plasmolysis treatment, P3N-PIPO-YFP remained within the cell wall, whereas the mTalin-CFP-labeled cytoplasm detached from the cell wall (Figure 3C, plasmolyzed).

To determine if P3N-PIPO colocalizes with the CI protein at PD, P3N-PIPO-GFP was coexpressed with CI-mRFP. At 48 hrs post-infiltration, the CI-mRFP punctate fluorescence predominantly overlapped the P3N-PIPO fluorescence at PD (Figure 3D, panel I). At 72 hrs, the size of P3N-PIPO-derived structures remained the same, whereas the PD-localized fluorescence of CI-mRFP expanded as thin thread-like structures into the cytoplasm (Figure 3D, panels II, III). The majority of the red fluorescent threads of CI-mRFP were seen to extend from P3N-PIPO-labeled spots (Figure 3D, panels II, III). These results suggest that CI is targeted to PD in the presence of P3N-PIPO, colocalizes with P3N-PIPO at PD, and subsequently forms PD-rooted thread-like structures.

The relocalization of CI-mRFP to PD in the presence of P3N-PIPO might suggest an interaction between CI and P3N-PIPO. To test this hypothesis, a bimolecular fluorescence complementation (BiFC) assay was carried out. CI-YN and P3N-PIPO-YC hybrid proteins, as well as the reverse hybrid combination, were coexpressed in *N. benthamiana* leaf cells. Strong BiFC fluorescence from both hybrid combinations was detected along the cell wall, highlighted by PD fluorescence that coincided with PD-associated PDLP1-CFP 48 hrs post-infiltration (Figure 3E, panels I, II). No BiFC fluorescence was detected in the negative control samples expressing combinations with non-hybrid YC or YN (data not shown). Taken together these data suggest that P3N-PIPO is a PD-located protein and may direct targeting of CI to PD through protein-protein interaction.

### PD targeting of P3N-PIPO and CI requires a functional secretory pathway and is independent of the actomyosin motility system

**Figure 4:**
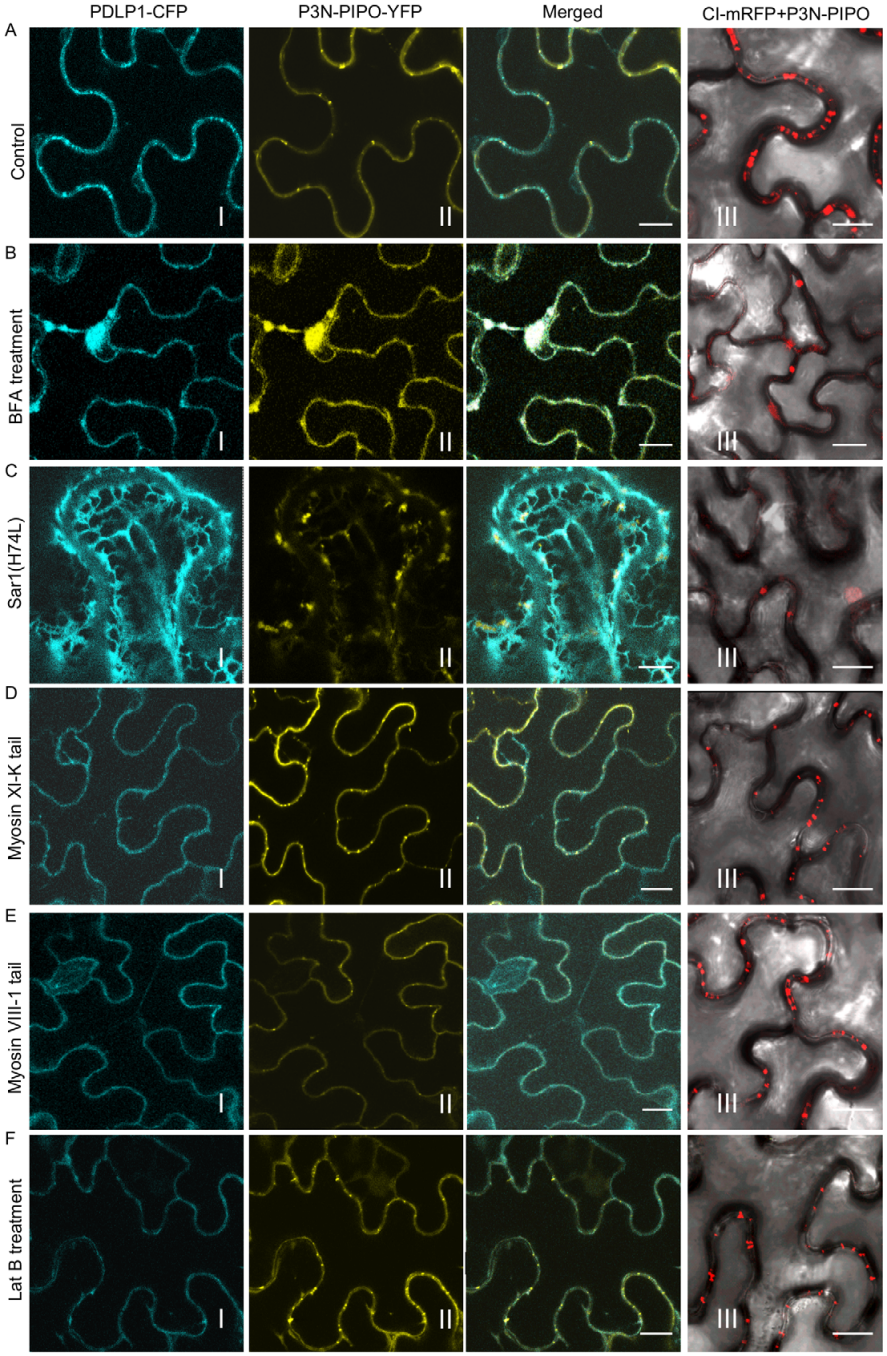
Targeting of P3N-PIPO and CI to PD requires the BFA-sensitive secretory pathway and is independent of the acto-myosin motility system. PD marker PDLP-1-CFP (panels I), P3N-PIPO-YFP (panels II) and CI-mRFP in the presence of untagged P3N-PIPO (panels III) were transiently expressed in leaf cells treated with water (Control, A), 50 µg/mL BFA (BFA, B), co-agroinfiltrated with the untagged COPII mutant Sar1(H74L) [Sar1(H74L), C], co-agroinfiltrated with the untagged myosin XI-K tail (Myosin XI-K tail, D), co-agroinfiltrated with the untagged myosin VIII-1 tail (Myosin VIII-1 tail, E), and 25 µM Lat B (Lat B, F). Images were taken 48 hrs post-agroinfiltration. N, nucleus. Bars, 10 µm.

Trafficking of plasmodesmatal proteins, such as PDLP1 (a type I membrane receptor-like protein) and ^C1^RGP (a class 1 reversibly glycosylated polypeptide) to PD has been shown to exploit the secretory pathway [32], [33]. Chemical or protein inhibitors were used to investigate the role of the secretory pathway in P3N-PIPO trafficking to PD. Both Brefeldin A (BFA) and a GTP-restricted mutant of the Sar1 protein [Sar1(H74L)] can block the ER-Golgi vesicular transport pathway [34]. In BFA-treated plant leaf cells, targeting of PDLP1-CFP or P3N-PIPO-YFP to PD was inhibited, with both PDLP1-CFP and P3N-PIPO-YFP accumulating in large compartments that are typical of BFA-treated cells (Figure 4A, B, panels I, II; Figure S2) [32]. Inhibition of PD localization of both proteins was incomplete (Figure S2), possibly due to only partial disruption of trafficking or to residual localization and accumulation prior to drug treatment. Consistent with recent findings [32], coexpression of non-tagged Sar1(H74L) caused the retention of PDLP1-CFP in the ER network (web-like fluorescence pattern) and inhibited the trafficking of PDLP1-CFP to PD (Figure 4A, C, panels I; Figure S2). In contrast, when coexpressed with Sar1(H74L), P3N-PIPO trafficking to PD was also inhibited and P3N-PIPO-YFP was cytosolic in the cells (Figure 4A, C, panels II; Figure S2). This is in agreement with the result that no typical transmembrane domains were found in TuMV P3N-PIPO using computer-assisted prediction tools described in Material and Methods. Of 100 cells observed under the confocal microscope, approximately 20% of punctate structures of PDLP1-CFP and P3N-PIPO-YFP were PD-located after BFA treatment or coexpression with untagged Sar1(H74L) (Figure S2). It is therefore concluded that, like some other PD-localized proteins, P3N-PIPO utilizes the secretory pathway for delivery to PD.

The actomyosin motility system, empowered by myosin motors, has been implicated in the targeting of viral MP proteins to PD [30] and intercellular spread of some plant viruses [35]. Higher plants possess two groups of myosins, class VIII and class XI [27]. The tails of myosins XI-K and VIII-1, which behave as dominant-negative antagonists to myosin function, were used to examine the role of myosins in the targeting of P3N-PIPO to PD. Interestingly, while overexpression of the tails inhibited targeting of PDLP1-CFP to PD, they had no apparent effects on P3N-PIPO-YFP steady-state accumulation patterns (Figure 4A, D, E, panels I, II; Figure S2). As the requirement of myosin motors for intracellular trafficking implies the utilization of the actin microfilaments, we further tested the effect of Latrunculin B (Lat B), an inhibitor of actin polymerization, on the targeting of P3N-PIPO to PD. Lat B treatment did not affect the targeting of P3N-PIPO to PD (Figure 4A, F, panel II; Figure S2). These data suggest that the actomyosin motility system, which is involved in the trafficking of plasmodesmatal proteins PDLP1 and some other viral MPs to PD, is not involved in PD-localization of P3N-PIPO.

The effect of secretory pathway inhibitors and dominant-negative mutants on the localization of CI was investigated. In the absence of P3N-PIPO or TuMV infection, none of these inhibitors interfered with the formation of CI-mRFP aggregates in the cytoplasm (data not shown). Consistently, analysis of the TuMV CI sequence with several computer-assisted prediction tools described in Material and Methods did not reveal any typical membrane domains. However, both BFA treatment and overexpression of Sar1(H4L) inhibited PD-localization of CI-mRFP in the presence of P3N-PIPO (Figure 4A, B, C, panels III; Figure S2). The targeting of CI-mRFP to PD was not obviously affected in leaf cells expressing the myosins tails or treated with LatB (Figure 4D, E, F, panels III; Figure S2). Thus, PD targeting of P3N-PIPO or CI (in the presence of P3N-PIPO) requires a functional secretory pathway and is independent of the actomyosin motility system.

### TuMV CP associates with PD-located CI structures during virus infection

**Figure 5:**
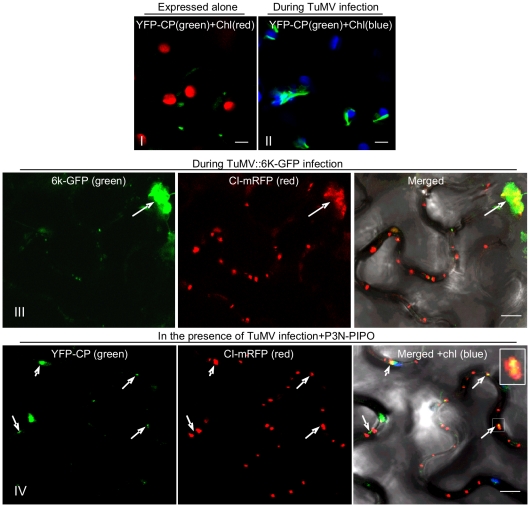
Association of TuMV CP with CI in TuMV-infected *N. benthamiana* leaf cells. (Panels I, II) TuMV CP tagged by YFP (YFP-CP) in the cytoplasm when expressed alone (panel I) or during virus infection (panel II). (Panel III) When coexpressed with the recombinant TuMV::6K-GFP infectious clone, some mRFP-CP is also present in proximity to the 6K-GFP-labeled replication complex (arrow). (Panel IV) TuMV YFP-CP attachment to PD-associated CI structures.(arrows; Inset) in the cell periphery in the presence of P3N-PIPO during virus infection. All images are taken 48 hrs post-agroinfiltration. Chl, chloroplasts. Bars, 8 *µ*m.

The assembled virion is likely the form in which potyviruses move through PD [25]–[28]. To assess the subcellular distribution of virions relative to the CI protein during virus infection, YFP-CP and CI-mRFP were coexpressed in *N. benthamiana* leaf cells infected with a recombinant TuMV-GFP infectious clone, or a TuMV infectious clone expressing a 6K-GFP fusion protein (TuMV::6K-GFP) [36], [37]. As described earlier, CI trafficked to PD and formed aggregates in the cytoplasm at 48 to 72 hrs post-agroinfiltration (Figure 2A). The fibrillar structures of YFP-CP were also observed either in the cytoplasm or along the cell wall (Figure 5). In the cytoplasm, CI aggregates are often associated with 6K vesicles where virus replication takes place (Figure 5, panel III) [37]. The fibrillar structures of CP represent virions or CP-containing nucleoprotein (NP) complexes [28]. In the cytoplasm, the fibrillar structures of CP were in close proximity to chloroplasts during virus infection (Figure 5, panel II). This was in contrast to CP punctate structures forming away from chloroplasts when expressed alone (Figure 5, panel I). Moreover, the fibrillar structures of YFP-CP were associated with PD-localized structures of mRFP-CI in the presence of P3N-PIPO during virus infection (Figure 5, panel IV), consistent with published results that the CP of *Pea seed-borne mosaic virus* (PSbMV) is present in the CI conical structures adjacent to PD [27], [28] and that TEV CP is required for cell-to-cell movement [25], [26].

### PD targeting of CI is essential for potyvirus cell-to-cell movement

**Figure 6:**
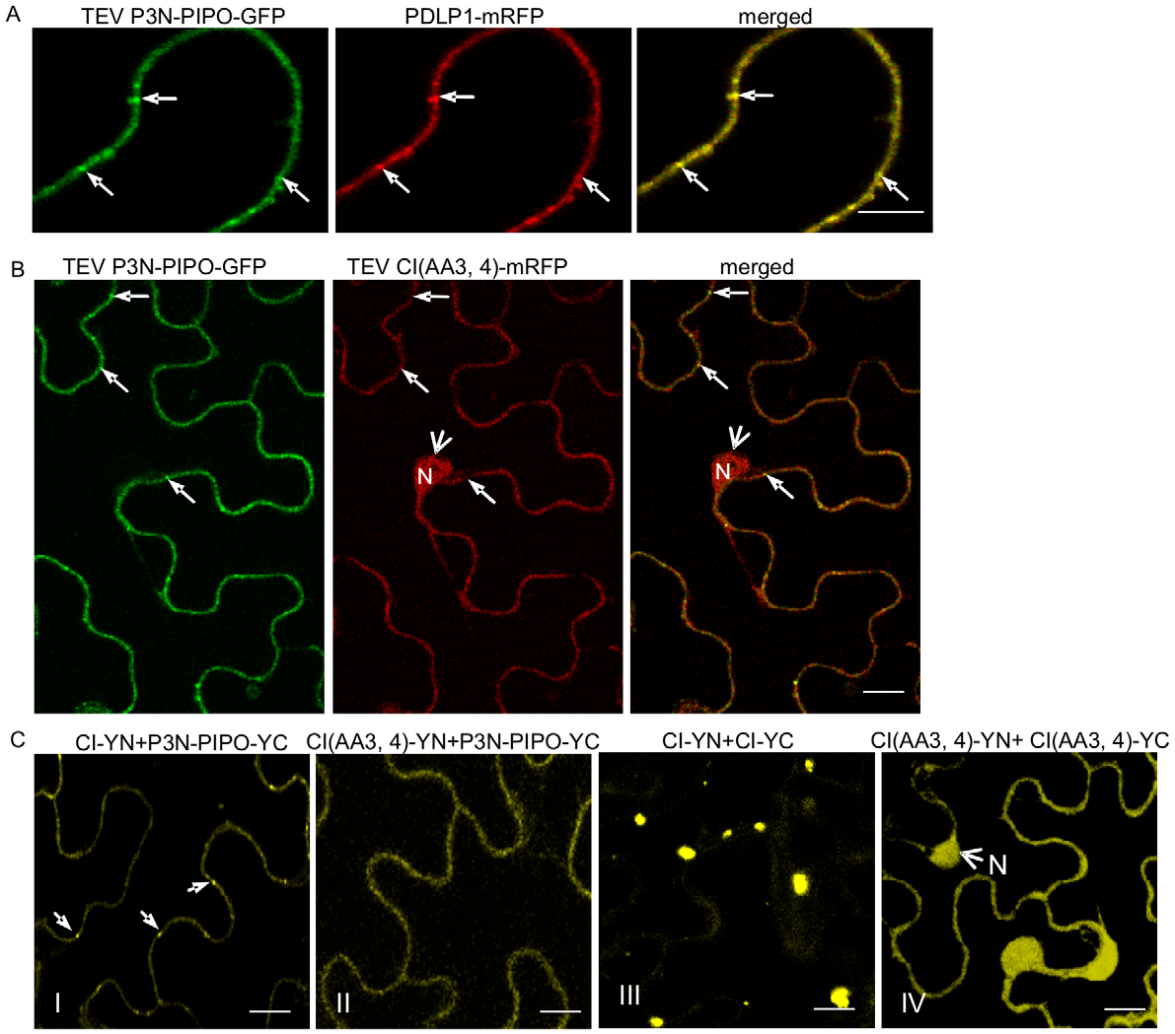
Subcellular localization of TEV P3N-PIPO and its interaction with TEV CI and the intercellular movement-defective mutant CI(AA3,4). (A) Colocalization of TEV P3N-PIPO-GFP with the PD marker PDLP1-mRFP. Arrows point to the PD-located P3N-PIPO-GFP and PDLP1-mRFP. (B) Coexpression of TEV CI(AA3,4)-mRFP does not change PD-localization of TEV P3N-PIPO-GFP. Arrows indicate PD-located P3N-PIPO-GFP. Arrowheads indicate nucleus-localization of TEV CI(AA3,4)-mRFP. (C) BiFC analysis of interactions of TEV CI-YN and TEV P3N-PIPO-YC (panel I), TEV CI (AA3, 4)-YN and TEV P3N-PIPO-YC(panel II), TEV CI-YN and TEV CI-YC (panel III), and TEV CI (AA3, 4)-YN and TEV CI (AA 3, 4)-YC. N, nucleus. Bars, 10 µm.

**Figure 7:**
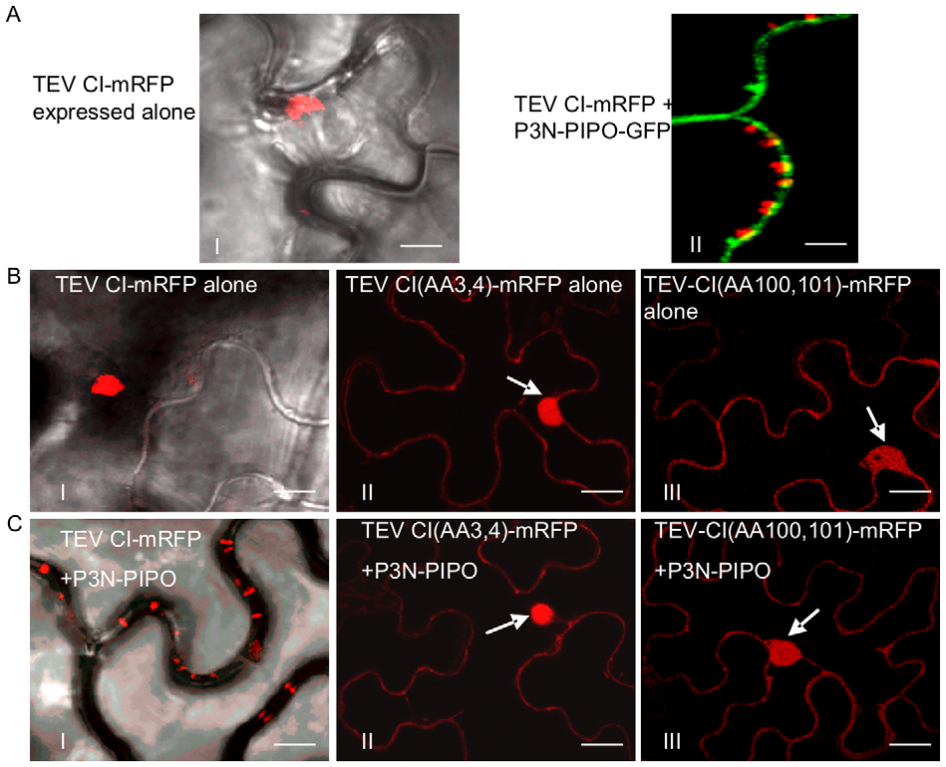
Two TEV CI mutants defective in cell-to-cell movement fail to form either cytoplasmic inclusions (when expressed alone) or PD-associated structures (in the presence of P3N-PIPO). (A) TEV CI-mRFP forms aggregates in the cytoplasm when expressed alone (panel I) and punctate spots along the cell walls when coexpressed with P3N-PIPO-GFP (panel II). (B) When expressed alone, TEV CI(AA3, 4)-mRFP (panel II) and TEV CI(AA100,101)-mRFP (panel III) are distributed in the nucleus and in periphery rather than forming typical inclusions in the cytoplasm (panel I). (C) In the presence of P3N-PIPO, TEV CI(AA3, 4)-mRFP (panel II) and TEV CI(AA100,101)-mRFP (panel III) are distributed in the nucleus and cell periphery rather than targeting PD (panel I). All images are taken 48 hrs post-agroinfiltration. Bars, 8 µm.

To determine if the P3N-PIPO protein of potyviruses other than TuMV direct targeting of their respective CI to PD, P3N-PIPO and CI of TEV were analyzed in *N. benthamiana* cells. Consistent with observations described above for TuMV, TEV P3N-PIPO colocalized with PDLP1 at PD (Figure 6A). TEV CI-mRFP formed aggregates in the cytoplasm when expressed alone (Figure 7A, panel I) and was targeted to PD with associated thread-like structures when coexpressed with TEV P3N-PIPO-GFP (Figure 7A, panel II).

Previous genetic analysis of TEV identified CI mutants with substitutions affecting two aspartic residues at positions 3 and 4, or two positively charged amino acids (lysine and argentine) at positions 100 and 101, with defects in cell-to-cell movement [9]. These mutants amplified to levels equivalent to that of the parental virus in isolated cells. When expressed alone or coexpressed with TEV P3N-PIPO, the two TEV CI mutant proteins, CI(AA3, 4)-mRFP and CI(AA100,101)-mRFP, were observed in the nucleus and cell periphery, but not in punctate PD or late-forming cytoplasmic aggregate patterns typical for the parental CI-mRFP protein (Figure 7B, C). Furthermore, coexpression of CI(AA3,4)-mRFP with P3N-PIPO-GFP did not affect the targeting of P3N-PIPO-GFP to PD (Figure 6B). BiFC experiments were performed to examine if TEV P3N-PIPO interacted with CI(AA3,4). As expected, the BiFC fluorescence resulting from parental CI-YN and P3N-PIPO-YC, or their reverse hybrid combination, was localized to PD (Figure 6C, panel I). Such targeting was compromised in cells with CI(AA3, 4)-YN and P3N-PIPO-YC or their reverse hybrid combination (Figure 6C, panel II). Though the mutated CI did interact with P3N-PIPO, the BiFC signal was evident in the cell periphery but not within distinct, punctate foci indicative of PD (Figure 6C, panel II). BiFC experiments also localized the self-interacting TEV CI mutant CI(AA3, 4) to the nucleus and cell periphery (Figure 6C, panel IV) rather than to typical inclusions in the cytoplasm (Figure 6B, panel III). BiFC fluorescence was not detected in cells expressing non-hybrid YN and YC proteins (data not shown). The lack of PD-localization of the mutant TuMV and TEV CI proteins may explain the cell-to-cell movement defects reported previously [9].

## Discussion

In the present study, TuMV CI was localized to the cell wall at the early stages of infection (Figure 2B, panel I; Figure 1A, panel II), and to thread-like structures emanating from punctate bodies (Figure 2B, panel II). CI structures disassociated from the cell wall and accumulated as aggregates in the cytoplasm at later time points (Figure 2A, panel III). By contrast, when expressed alone, CI-mRFP only aggregated in the cytoplasm (Figure 2A, panel I). P3N-PIPO was found to direct the CI fusion protein to the cell wall-associated punctate bodies, which colocalized with PD protein marker PDLP1 (Figure 2C, D; Figure 3). Significantly, the CI protein from multiple potyviruses was shown to associate with P3N-PIPO, and target to PD in a P3N-PIPO-dependent manner (Figure 2; Figure 7). This is consistent with several ultrastructural studies showing that the CI protein of *Sorghum mosaic virus* (Figure S3), TVMV [27] and *Wheat spindle streak mosaic virus* [38] form conical structures adjacent to PD, and possibly traverse the PD in a fine thread-like structure. It is worth noting that polyclonal antibodies against TVMV P3 specifically labeled cell wall-bound deposits of CI in TVMV-infected cells [27], [39]. Since TVMV P3N-PIPO and P3 share an N-terminal region of approximately 20 kDa [20], the P3 polyclonal antibodies may actually have recognized P3N-PIPO. This speculation is also supported by the finding that potyviral P3 protein does not interact with CI [40]. These data point to a role for P3N-PIPO directing CI to PD, anchoring the CI proteins therein and/or facilitating the deposition of CI through PD.

We show that P3N-PIPO is a PD-located protein and interacts with CI (Figure 3). Unlike PDLP1, both P3N-PIPO and CI lack a typical transmembrane domain. Disrupting the ER-Golgi secretory pathway obstructed the targeting of P3N-PIPO and CI to PD, whereas impairing the actomyosin motility system had little effect on their PD localization (Figure 4). Thus, targeting of P3N-PIPO and CI to PD requires a functional secretory pathway but not the actomyosin motility system. Interestingly, targeting of PDLP1 to PD is mediated by a transmembrane domain and requires both the ER-Golgi secretory pathway and the actomyosin motility system (Figure 4) [32]. It appears that some viral MPs, i.e., *Beet yellow virus* MP, require the actomyosin motility system for their PD targeting, while others, such as *Tobacco mosaic virus* MP, do not [30]. Since targeting of P3N-PIPO and PDLP1 to PD shares the BFA-sensitive secretory pathway and differs in the requirement of the actomyosin motility system (Figure 4), exactly how the secretion route branches from the ER-Golgi pathway to PD is yet to be determined.

The delivery of CI-mRFP to PD was mediated by P3N-PIPO in a ratio dependent manner. As P3N-PIPO is translated via ribosomal frameshifting [20], the ratio of CI to P3N-PIPO biosynthesis depends on the frameshift efficiency. In PSbMV–infected pea cotyledons, cell wall-associated CI conical structures were found only in the infection front where active genome translation and replication was occurring [28]. In cells behind this zone, CI was no longer associated with the cell wall, which is consistent with the temporal series of events documented in the current study. The transient association of CI structures with PD could be conditional upon the level of viral genome translation, frameshifting and the subsequent CI:P3N-PIPO ratio.

Targeting of CI to PD by P3N-PIPO may also be affected by CI self-interactions. Two movement-defective CI mutants (DD3, 4AA and KK101,102AA), previously shown to have reduced self-interacting strength [41], lost ability to form cytoplasmic structures (Figure 6C, panel III; Figure 7B). When these mutants were either expressed alone or in the presence of P3N-PIPO, they were distributed to the cell periphery and the nucleus (Figure 7B,C). Though these mutants could still interact with P3N-PIPO (Figure 6C), they were unable to accumulate at PD to form the thread-like structure (Figure 7C). Therefore, the proper self-assembly of CI is necessary for its interaction with P3N-PIPO to form the cone-shape structure at PD for viral cell-to-cell movement.

**Figure 8:**
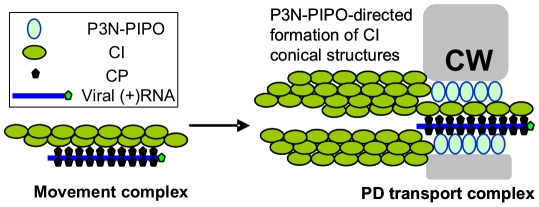
Model for potyvirus intercellular transport through PD. The virion-CI movement complex is intracellularly transported to the modified PD where CI forms conical structures anchored by the PD-located P3N-PIPO. The virion is then fed through the CI structures and PD to enter the adjacent cell. CW, cell wall.

During the reviewing process of this paper, Wen and Hajimorad reported that mutation of the putative SMV PIPO impedes cell-to-cell movement [42], providing genetic evidence that P3N-PIPO is a potyviral MP. Thus, our data reveals the mechanism by which P3N-PIPO functions as an MP. Taking into account these new findings, we propose a model for the formation of movement complexes that facilitate intercellular transport of potyviruses (Figure 8). Initial events likely involve the recruitment of nascent virus particles by CI or self-interacting CI structures at membrane-bound sites of replication adjacent to chloroplasts. Next, CI-virion complexes may associate with either pre-targeted P3N-PIPO followed by trafficking to PD, or with PD-associated P3N-PIPO. CI structures are then proposed to grow from P3N-PIPO-anchored sites at PD, forming thread-like structures that might recruit additional virus particles for transport. Actual movement of virus particles through PD may be facilitated by PD-traversing CI complexes, although the details of this proposed event are not known. It is difficult to imagine a mechanistic role for the late-forming cytoplasmic aggregates in virus movement, as these form during a post-movement phase of infection. This model bears some similarity to that proposed by Jackson and colleagues for cell-to-cell movement of hordeiviruses [3], [4], which encode three MPs (TGB1, TGB2 and TGB3). The hordeivirus model states that TGB1 binds the viral RNAs to form movement complexes. Interaction with TGB3 targets the TGB1-RNA movement complex to PD. TGB2 then interacts with TGB3 to stabilize the movement complex at PD and facilitate PD gating. The key deficits of the proposed models center around the mechanisms underlying gating, the mechanisms to generate force, and the impact of these virus-associated events on the normal functions of PD in intercellular trafficking and defense. These represent critical areas of future study.

## Materials and Methods

### Plasmid construction

Gateway technology (Invitrogen, Burlington, Ontario, Canada) was used to generate all plasmid clones used in this work. Gene sequences were amplified by PCR using Phusion DNA polymerase (NEB). The resulting DNA fragments were purified and transferred by recombination into the entry vector pDONR221 (Invitrogen) using BP clonase II (Invitrogen) following the supplier's recommendations. Insertions in the resulting pDONR clones were verified by DNA sequencing. Destination vectors pEarleygate100, pEarleygate102, pMDC83, pMDC43, pGWB454 and pGWB455 were used to express untagged protein, C-terminal CFP-HA fusion protein, C-terminal GFP-6xHis fusion protein, N-terminal GFP fusion protein, C-terminal mRFP fusion protein, and N-terminal mRFP fusion protein, respectively [43]–[46]. The vectors used in the BiFC assay were modified from pEarlyGate vectors 201 and 202, where the YFP N-terminus comprises amino acids 1–174 and the C-terminus comprises amino acids 174–239 [36]. The recombinant TuMV infectious clones containing 6K-GFP (TuMV:6K-GFP) and GFP (TuMV:GFP) were as described previously [37], [47]. Plasmid pTEV7DA-GFP was described [48]. The P1, HC-Pro, P3, P3N-PIPO, 6K1, CI, 6K2, NIa, NIb and CP coding regions of TuMV were amplified by PCR, recombined into pDONR221, and then into the binary destination vector pEarleygate100 for expressing nontagged proteins. The entry vector pDONR221 containing TuMV P3N-PIPO or CI were transferred by recombination into the binary destination vectors pEarleygate102, pMDC83 and pGWB454 to produce plasmids TuMV P3N-PIPO-CFP, TuMV P3N-PIPO-GFP, TuMV P3N-PIPO-mRFP, TuMV CI-GFP, and TuMV P3N-PIPO-mRFP. The entry vector pDONR221 containing TuMV CP was transferred by recombination into the binary destination vectors pMDC43 and pGWB455 to produce plasmids TuMV GFP-CP and TuMV mRFP-CP, respectively. The TEV P3N-PIPO, wild-type CI, and two CI mutants (DD3, 4AA and KR100,101AA) were amplified by PCR, recombined into pDONR221, and then into the binary destination vectors pEarleygate100, pMDC43 or pGWB455 to generate plasmids TEV P3N-PIPO (nontagged), TEV P3N-PIPO-GFP, TEV-CI-mRFP, TEV CI(AA3, 4)-mRFP and TEV CI(AA100,101)-mRFP. For BiFC experiments, the pDONR221vectors containing TuMV P3N-PIPO and CI were recombined into BiFC vectors YN and YC to generate P3N-PIPO-YN (YC) and CI-YN (YC), respectively. The pDONR221 vectors containing TEV P3N-PIPO, CI, CI(AA3, 4) and CI(AA100,101) were recombined into BiFC vectors YN and YC to generate TEV P3N-PIPO-YN (YC), CI-YN (YC), CI(AA3, 4)-YN (YC) and CI(AA100,101)-YN (YC), respectively. The plasmid containing the PDLP1:GFP was a kind gift from Andrew J. Maule (John Innes Centre, UK). The DNA fragment for PDLP1 was amplified and recombined into pDONR221 and then into the binary destination vector pGWB454 to give PDLP1-mRFP. The construct for expressing GFP-REM was kindly provided by Sébastien Mongrand (Centre National de la Recherche Scientifique-University of Bordeaux, France) [23]. The construct for expressing the myosins XI-K and VIII-1 tails were kindly provided by Valerian V. Dolja (Oregon State University) [30], [49]. The construct for expressing the actin filament marker mTalin-CFP was previously described [37].

### Transient expression in *N. benthamiana*

Binary vectors were transformed into *Agrobacterium tumefaciens* GV3101. For agroinfiltration, agrobacterial cultures were grown overnight in LB containing appropriate antibiotics. The agrobacteria were collected by centrifugation, and then resuspended in 10 mM MgCl_2_ containing 100 µM acetosyringone. After a minimum of 2 h incubation at room temperature, the culture was diluted to an optical density of 0.2–0.5 at 600 nm (OD_600_). *N. benthamiana* plants were agroinfiltrated with appropriate agrobacterial cultures and the agroinfiltrated plants were maintained under normal growth conditions for 2 to 4 days. For plasmolysis, plant tissue was infiltrated with 30% glycerol and viewed immediately.

For co-agroinfiltration assays with different ratio of CI and P3N-PIPO expression plasmids, different volumes of agrobacterial cells (OD_600_ = 0.6) containing expression plasmids untagged TuMV P3N-PIPO, TuMV P3N-PIPO-6xHis, TuMV CI-mRFP or TuMV CI-YFP-HA were mixed to achieve desired ratios of CI to PIPO. The total cell population of each mixture was kept constant for each infiltration experiment. Co-agroinfiltration of different plasmid combinations, i.e., TuMV CI-mRFP and TuMV P3N-PIPO or TuMV CI-YFP-HA and TuMV P3N-PIPO-6xHis was repeated at least three times. For western blot analyses, leaf samples were examined under the confocal microscope to confirm the subcellular localization of the fluorescent fusion proteins. Total proteins were extracted from *N. benthamiana* leaves agroinfiltrated with mixtures of agrobacterial cells containing TuMV CI-YFP-HA and TuMV P3N-PIPO-6xHis. Immunoblot was conducted with anti-HA IgG (Sigma, St. Lous, MO) and anti-His IgG (Abcam, Cambridge, MA) following the protocols recommended by the suppliers.

### Inhibitor studies

Inhibitor studies were done following transient coexpression of PDLP1-CFP and P3N-PIPO-YFP or coexpression of CI-mRFP and untagged P3N-PIPO in 4-week-old *N. benthamiana* plants. The infiltrated plant leaves were infused with BFA (50 µg/ml in water) at 40 h post-infiltration with the expression constructs and viewed 12 h post-BFA treatment. For the disruption of actin, leaf tissues were treated with 25 µM Lat B (Sigma-Aldrich, Oakville, Ontario, Canada) for 2 h. Negative controls were run in parallel.

### Confocal microscopy

Plant tissue was imaged at room temperature using a Leica TCS SP2 inverted confocal microscope with an Argon ion laser. GFP was excited at 488 nm and the emitted light was captured at 505 to 555 nm. Light emitted at 630–680 nm was used to record chlorophyll autofluorescence. YFP was excited at 514 nm and captured at 525-650 nm. mRFP was excited at 543 nm and captured at 590–630 nm. Images were captured digitally and handled using the Leica LCS software.

### Sequence sources and analyses

The TEV and TuMV genome sequences used in this study were retrieved from GenBank (http://www.ncbi.nlm.nih.gov/Genbank/) with accession numbers DQ986288.1 and EF028235.1, respectively. Transmembrane helices in the P3, P3N-PIPO and CI proteins of TuMV were predicted as previously described [50].

## Supporting Information

Figure S1 Western blot analysis of proteins extract from *N. benthamiana* leaf tissues agroinfiltrated with various ratios of agrobacterial cells containing plasmid CI-YFP-HA or P3N-PIPO-6xHis in with anti-HA IgG and anti-His IgG based on standard protocol. Fifty µgs of total proteins per lane was separated by 12.5% sodium dodecylsulphate-polyacrylamide gel electrophoresis (SDS-PAGE), transferred onto a polyvinylidene difluoride membrane and probed with antibodies against HA or His tags.
(0.78 MB TIF)

Figure S2 PD-located CI-mRFP (coexpressing with untagged P3N-PIPO), PDLP1-CFP and P3N-PIPO-YFP in *N. benthamiana* leaf cells treated with 50 µg/mL BFA or 25 µM Lat B, or coexpressing the untagged COPII mutant Sar1(H74L), the untagged myosin XI-K tail,or the untagged myosin VIII-1 tail. Values represent the mean number with SE that is given as a percentage relative to the control.
(0.46 MB TIF)

Figure S3 Electron micrographs showing that CI forms the conical structures at PD and enters a neighboring cell in a linear form (arrows) traversing the PD in *Sorghum mosaic virus*-infected plant tissue. CW, cell wall. Bars, 100 nm.
(2.02 MB TIF)
