# Supplementary figures and images for: Formation of Complexes at Plasmodesmata for Potyvirus Intercellular Movement Is Mediated by the Viral Protein P3N-PIPO
Source: PLoS Pathog. 2010 Jun 24;6(6):e1000962. doi: 10.1371/journal.ppat.1000962 (PMC2891837; doi:10.1371/journal.ppat.1000962)

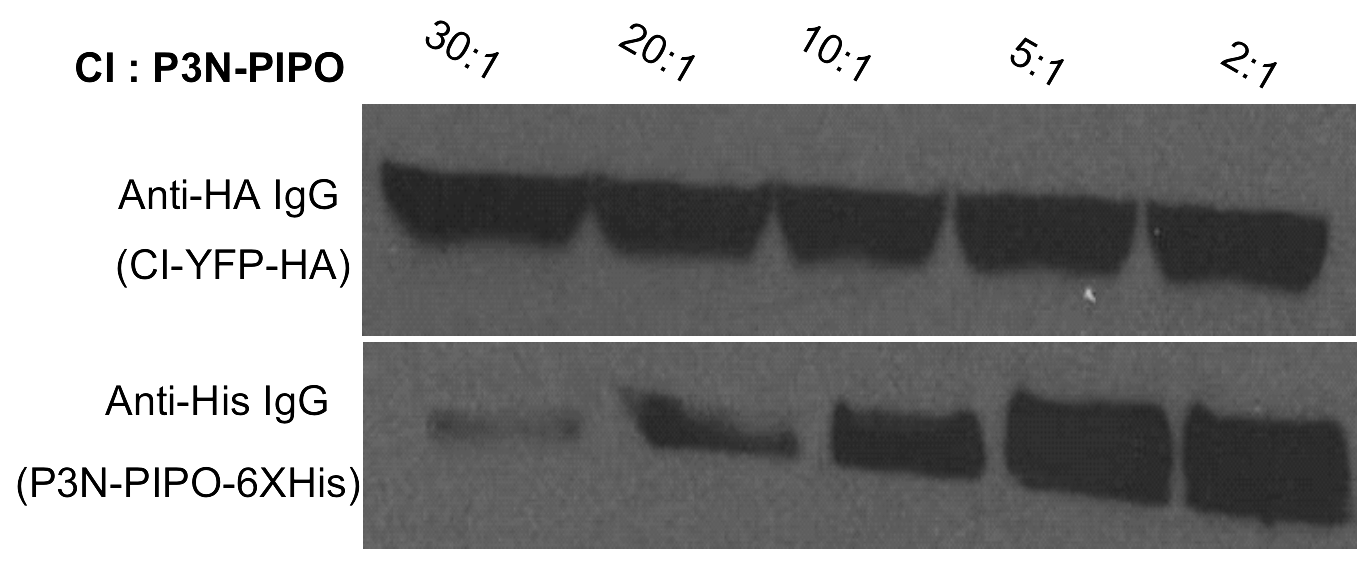

Supplement: Figure S1 — Western blot analysis of proteins extract from N. benthamiana leaf tissues agroinfiltrated with various ratios of agrobacterial cells containing plasmid CI-YFP-HA or P3N-PIPO-6xHis in with anti-HA IgG and anti-His IgG based on standard protocol. Fifty µgs of total proteins per lane was separated by 12.5% sodium dodecylsulphate-polyacrylamide gel electrophoresis (SDS-PAGE), transferred onto a polyvinylidene difluoride membrane and probed with antibodies against HA or His tags. (0.78 MB TIF) [file ppat.1000962.s001.tif]

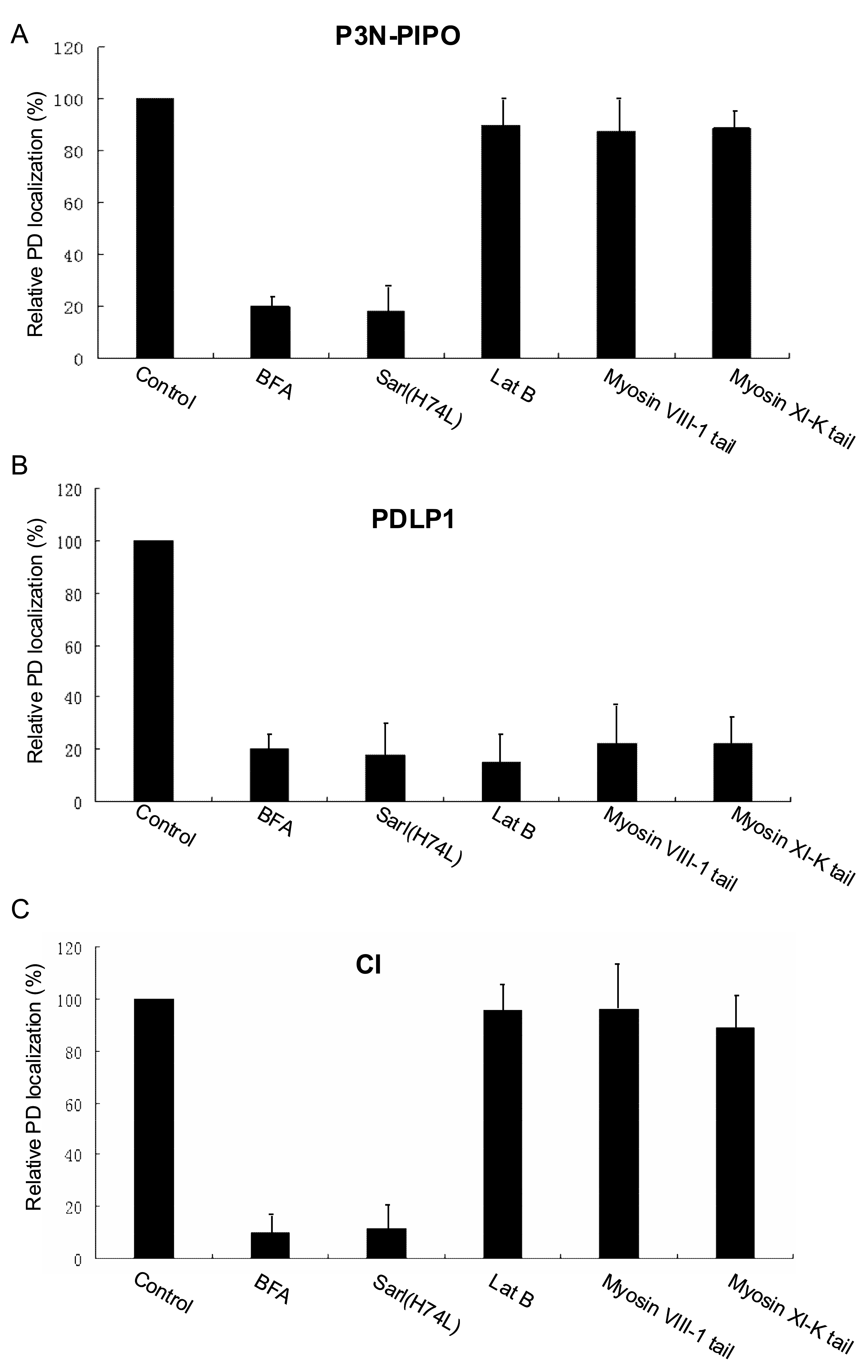

Supplement: Figure S2 — PD-located CI-mRFP (coexpressing with untagged P3N-PIPO), PDLP1-CFP and P3N-PIPO-YFP in N. benthamiana leaf cells treated with 50 µg/mL BFA or 25 µM Lat B, or coexpressing the untagged COPII mutant Sar1(H74L), the untagged myosin XI-K tail,or the untagged myosin VIII-1 tail. Values represent the mean number with SE that is given as a percentage relative to the control. (0.46 MB TIF) [file ppat.1000962.s002.tif]

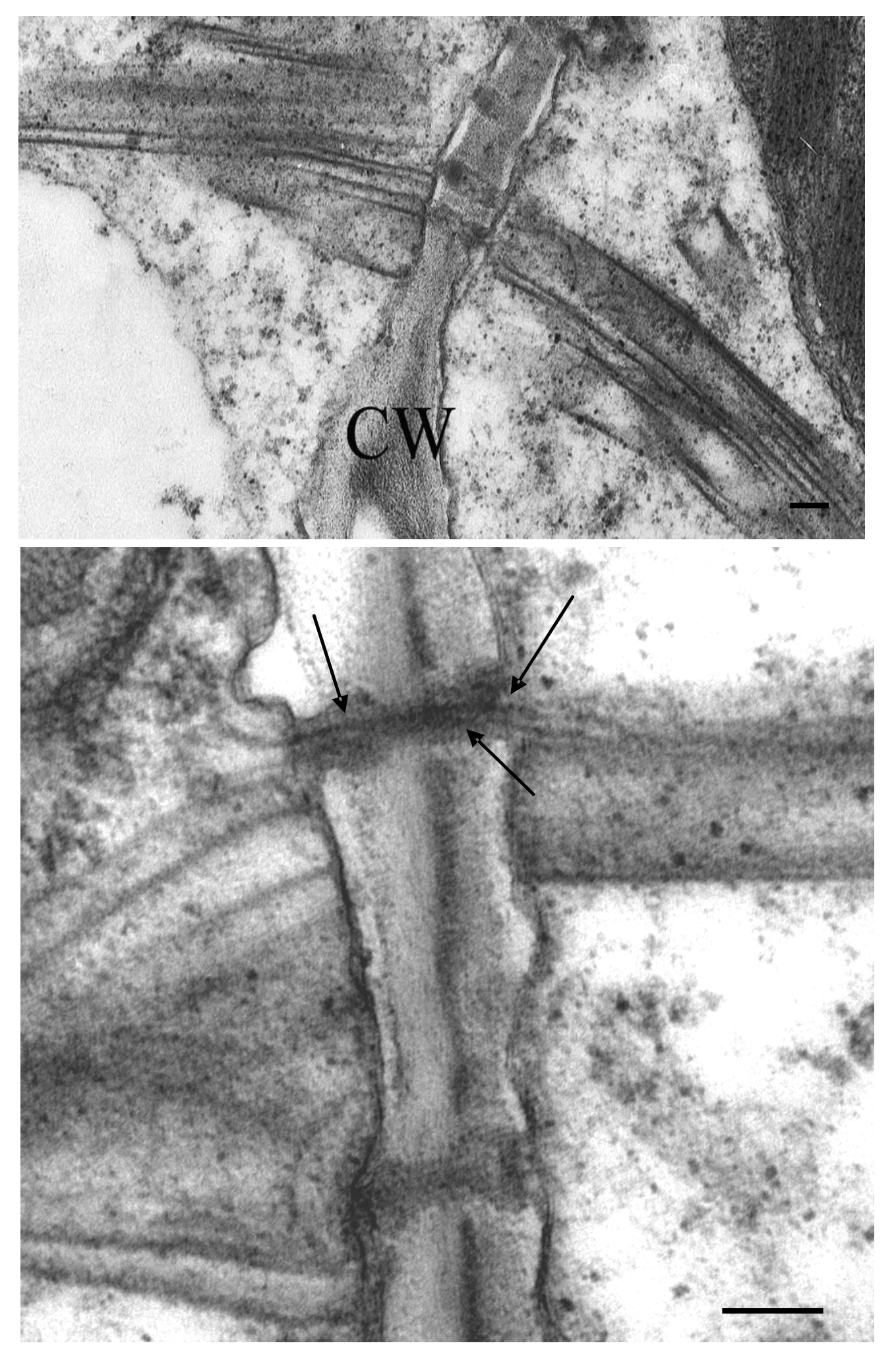

Supplement: Figure S3 — Electron micrographs showing that CI forms the conical structures at PD and enters a neighboring cell in a linear form (arrows) traversing the PD in Sorghum mosaic virus-infected plant tissue. CW, cell wall. Bars, 100 nm. (2.02 MB TIF) [file ppat.1000962.s003.tif]
